# External validity of a prognostic nomogram for locoregionally advanced nasopharyngeal carcinoma based on the 8th edition of the AJCC/UICC staging system: a retrospective cohort study

**DOI:** 10.1186/s40880-018-0324-x

**Published:** 2018-09-03

**Authors:** Pu-Yun OuYang, Kai-Yun You, Lu-Ning Zhang, Yao Xiao, Xiao-Min Zhang, Fang-Yun Xie

**Affiliations:** 1 Department of Radiation Oncology, Sun Yat-sen University Cancer Center, State Key Laboratory of Oncology in South China, Collaborative Innovation Center for Cancer Medicine, No. 651 Dongfeng East Road, Guangzhou, 510060 Guangdong P.R. China; 2 Department of Radiation Oncology, Sun Yat-sen Memorial Hospital, Sun Yat-sen University, Guangzhou, 510060 Guangdong P.R. China; 3 Department of Oncology, The First Affiliated Hospital of Guangdong Pharmaceutical University, Guangzhou, 510080 Guangdong P.R. China

**Keywords:** 8th AJCC/UICC staging system, Concurrent chemotherapy, Intensity-modulated radiotherapy, Nasopharyngeal carcinoma, Nomogram

## Abstract

**Background:**

The tumor–node–metastasis (TNM) staging system does not perform well for guiding individualized induction or adjuvant chemotherapy for patients with locoregionally advanced nasopharyngeal carcinoma (NPC). We attempted to externally validate the Pan’s nomogram, developed based on the 8th edition of the American Joint Committee on Cancer (AJCC)/Union for International Cancer Control (UICC) staging system, for patients with locoregionally advanced disease. In addition, we investigated the reliability of Pan’s nomogram for selection of participants in future clinical trials.

**Methods:**

This study included 535 patients with locoregionally advanced NPC who were treated between March 2007 and January 2012. The 5-year overall survival (OS) rates were calculated using the Kaplan–Meier method and compared with predicted outcomes. The calibration was tested using calibration plots and the Hosmer–Lemeshow test. Discrimination ability, which was assessed using the concordance index, as compared with other predictors.

**Results:**

Pan’s nomogram was observed to underestimate the 5-year OS of the entire cohort by 8.65% [95% confidence interval (CI) − 9.70 to − 7.60%, *P *< 0.001] and underestimated the 5-year OS of each risk group. The differences between the predicted and observed 5-year OS rates were smallest among low-risk patients (< 135 points calculated using Pan’s nomogram; which predicted minus observed OS, − 6.41%, 95% CI − 6.75 to − 6.07%, *P *< 0.001) and were largest among high-risk patients (≥ 160 points) (− 13.56%, 95% CI − 15.48 to − 11.63%, *P *< 0.001). The Hosmer–Lemeshow test suggested that the predicted and observed 5-year OS rates had no ideal relationship (*P *< 0.001). Pan’s nomogram had better discriminatory ability compared with the levels of Epstein–Barr virus DNA acid (EBV DNA) and the 7th or 8th AJCC/UICC staging system, although not better compared with the combination of EBV DNA and the 8th staging system. Additionally, Pan’s nomogram was marginally inferior to our predictive model, which included the 8th AJCC/UICC N-classification, age, gross primary tumor volume, lactate dehydrogenase, and body mass index.

**Conclusions:**

Pan’s nomogram underestimated the 5-year OS of patients with locoregionally advanced NPC at our cancer center, and may not be a precise tool for selecting participants for clinical trials.

## Background

Nasopharyngeal carcinoma (NPC) arises from the squamous cells of the epithelial lining of the nasopharynx. Radiotherapy is the primary treatment modality because of NPC’s confined anatomical location and high sensitivity to radiation. The non-specificity of nasal and aural symptoms accounts for locoregionally advanced disease in 70% of patients upon initial diagnosis [1]. Subsequently, these patients have a high risk of distant metastasis and mortality [2, 3] even if treated with concurrent chemoradiotherapy. Accordingly, induction chemotherapy is commonly administered before radiotherapy in clinical practice although randomized controlled trials have not yet contributed to a consensus about its survival benefit [4–8]. In addition, there are no effective adjuvant chemotherapy regimens that have been identified for these patients after radiotherapy [9–13]. Although the tumor, node and metastasis (TNM) staging system of the American Joint Committee on Cancer (AJCC)/Union for International Cancer Control (UICC) was the main tool used to identify patients in these clinical trials, however, the findings of these trials advocate that future clinical trials require more effective stratification method for the identification of high-risk patients, instead of enrolling every patient with locoregionally advanced NPC.

Pan et al. [14] have developed a nomogram comprising of patient’s age, gross primary tumor volume (GTVp), lactate dehydrogenase (LDH) level, and the 8th edition of the AJCC/UICC staging system [15, 16] using a population of 1197 patients treated at the Fujian Provincial Cancer Hospital. Its performance was tested in a cohort of 416 patients from Pamela Youde Nethersole Eastern Hospital, which achieved a concordance index (C-index) of 0.760 [95% confidence interval (CI), 0.723–0.796], which demonstrated significantly superior (*P *< 0.01) discriminatory power compared to the 8th AJCC/UICC staging system (C-index, 0.654; 95% CI, 0.622–0.686).

Although Pan’s nomogram may have greater potential than the 8th AJCC/UICC edition to identify patients for inclusion in clinical trials, however, since it was developed from a cohort of patients with stage I–IVa disease, its validity for specifically identifying patients with locoregionally advanced disease remains unknown. Additionally, external validation is important before clinical application to individualized randomized controlled trials of induction or adjuvant chemotherapy. As such, we first assessed Pan’s nomogram discriminatory accuracy and calibration by using a large external cohort of patients with stage III–IVb NPC who underwent intensity-modulated radiotherapy (IMRT) and concurrent chemotherapy alone. Second, we performed a direct comparison of its performance with that of Epstein–Barr virus deoxyribonucleic acid (EBV DNA), the most recent and potential biomarker for NPC [17], in an attempt to improve Pan’s nomogram.

## Methods

### Patient selection

Between March 2007 and January 2012, patients were deemed eligible for this study if they met the following inclusion criteria: (1) newly diagnosed with the World Health Organization type 2 or 3 NPC; (2) restaged to III–IVb (T1-2N2-3M0 and T3-4N0-3M0, based on the 8th edition of the AJCC/UICC staging system) according to pretreatment magnetic resonance imaging (MRI) of the nasopharynx and neck, chest radiography or computed tomography (CT), abdominal sonography or CT, a whole-body bone scan or [^18^F]-fluorodeoxyglucose positron emission tomography combined with computed tomography (PET/CT); (3) ages between 20 and 75 years old; (4) treated with IMRT plus concurrent chemotherapy alone; and (5) had pretreatment levels of EBV DNA and hemoglobin. Patients were excluded if they had received anticancer therapy prior to diagnosis at our hospital, were pregnant or lactating, or if they were diagnosed with synchronous/metachronous cancer lesion(s) before or during the treatment or follow-up period.

### Treatment

The cumulative radiation doses were administered in 30–33 fractions at ≥ 66 Gy to the primary tumor, ≥ 60 Gy to the involved neck area, and ≥ 50 Gy to potential sites of local infiltration and bilateral cervical lymphatics. Other IMRT information were similar to as previously detailed [18]. Concurrent chemotherapy was administrated with cisplatin/nedaplatin, 30–40 mg/m^2^ weekly for up to seven cycles or 80–100 mg/m^2^ every 3 weeks for two to three cycles.

### Follow up

Patients were followed at least once every 3 months during the first 3 years and every 6 months thereafter. Detailed recordings of history and physical examinations were performed at each follow-up visit. Nasopharyngoscopy with or without biopsy, MRI of the head and neck, chest radiography or CT, abdominal sonography or CT, a whole-body bone scan, or [^18^F]-fluorodeoxyglucose PET/CT were performed to detect locoregional relapse, distant metastasis, or both. Salvage treatment including reirradiation, surgery or chemotherapy, or both, was delivered to patients with confirmed relapse, distant metastasis, or persistent disease.

### Statistical analysis

The 5-year overall survival (OS) rate, defined from the date of treatment to death from any cause, was predicted using Pan’s nomogram for the entire cohort and each of the three different risk groups (low-risk, < 135 points; intermediate-risk, 135 to < 160 points; high-risk, ≥ 160 points calculated according to Pan’s nomogram) as suggested by Pan et al. [14]. The 5-year OS rate was calculated using the Kaplan–Meier method. We compared the observed and predicted 5-year OS rates using one-sample t test, where the predicted survival was served as the fixed variable while the observed value served as the assessed variable.

Next, we assessed the calibration of the model by plotting the observed and predicted 5-year OS outcomes and confirmed the findings using the Hosmer–Lemeshow calibration test [19]; for which a significant test statistic indicates that the model does not calibrate perfectly. Furthermore, discriminatory accuracy was assessed using Harrell’s concordance index (C-index) [20], where it is generally accepted that a higher C-index suggests a greater ability of the model to discriminate outcomes.

We compared the discriminatory accuracy of Pan’s nomogram vs EBV DNA levels, the 7th and 8th editions of the AJCC/UICC staging system, and the best predictive model of our dataset. To develop our best predictive model, prognostic factors such as age [21], sex [22], body mass index (BMI) [23], hemoglobin [24], and LDH [25], were included in backward multivariate Cox regression analysis. EBV DNA was categorized as previously described [26] because of its nonlinear effect detected using three-knot restricted cubic splines [27] nested within the Cox model.

Statistical analyses were performed using Stata version 14.1 (StataCorp LP, College Station, Texas, USA) and R version 3.3.1 (https://cran.r-project.org/). A two-sided *P *< 0.05 was considered as statistically significant.

## Results

### Patients

In total, 535 patients were found eligible for this study. Table 1 lists the comparisons between our cohort and the Fujian Provincial Cancer Hospital cohort for which our analysis was restricted to patients with locoregionally advanced NPC who received IMRT plus concurrent chemotherapy treatment alone. This study results demonstrated significant differences in tumor stages and modes of chemotherapy between the two cohorts. Also, the patients from our cohort had a lower mean level of LDH (171.3 vs 193.4 U/L).

**Table 1:** Comparison of the different characteristics between patients from the Sun Yat-sen University Cancer Center and those from the Fujian Provincial Cancer Hospital’s cohort [14]

Characteristics	Sun Yat-sen University Cancer Center patients cohort *n* (%)	Fujian Provincial Cancer Hospital cohort *n* (%)
Total	535	1197
Age (years)^a^		
Median (range)	45 (20–72)	46 (11–84)
Mean	45.4	46.4
The 8th AJCC/UICC clinical stage [cases (%)]^a^		
III	421 (78.7)	381 (31.8)
IVa–b	114 (21.3)	462 (38.6)
GTVp (cm^3^)^a^		
Median (range)	33.8 (2.6–165.2)	32.8 (0.1–235.6)
Mean	41.0	41.2
LDH (U/L)^a^		
Median (range)	165.4 (101.8–448.6)	183 (106–751)
Mean	171.3	193.4
Sex		
Male	382 (71.4)	905 (75.6)
Female	153 (28.6)	292 (24.4)
Histology^b^		
II	27 (5.0)	51 (4.3)
III	508 (95.0)	1134 (94.7)
The 8th AJCC/UICC T-classification		
T1	22 (4.1)	285 (23.8)
T2	45 (8.4)	220 (18.4)
T3	389 (72.7)	294 (24.6)
T4	79 (14.8)	398 (33.2)
The 8th AJCC/UICC N-classification		
N0	73 (13.6)	174 (14.5)
N1	268 (50.1)	658 (55.0)
N2	149 (27.9)	270 (22.6)
N3	45 (8.4)	95 (7.9)
The 7th AJCC/UICC T-classification		
T1	22 (4.1)	NA
T2	43 (8.0)	NA
T3	325 (60.7)	NA
T4	145 (27.1)	NA
The 7th AJCC/UICC N-classification		
N0	73 (13.6)	NA
N1	273 (51.0)	NA
N2	162 (30.3)	NA
N3a	11 (2.1)	NA
N3b	16 (3.0)	NA
The 7th AJCC/UICC clinical stage		
III	367 (68.6)	NA
IVa	141 (26.4)	NA
IVb	27 (5.0)	NA
EBV DNA (10^3^ copies/mL)^a^		
Median (range)	1.65 (0–12,600)	NA
Mean	97.9	NA
EBV DNA (copies/mL)^c^		
< 10^3^	246 (46.0)	NA
10^3^–10^4^	120 (22.4)	NA
10^4^–10^5^	110 (20.6)	NA
10^5^–10^6^	52 (9.7)	NA
≥ 10^6^	7 (1.3)	NA
Hb (g/L)		
Median (range)	143.0 (88.0–183.0)	143 (80–171)
Mean	141.6	143
BMI (kg/m^2^)		
Median (range)	23.0 (15.2–39.7)	NA
Mean	23.1	NA
Chemotherapy		
None	0 (0.0)	181 (15.1)
Concurrent	535 (100.0)	NA
Other	0 (0.0)	NA

*AJCC* American Joint Committee on Cancer, *UICC* Union for International Cancer Control, *GTVp* gross primary tumor volume, *LDH* lactate dehydrogenase, *NA* not available, *EBV DNA* Epstein–Barr virus deoxyribonucleic acid, *Hb* hemoglobin, *BMI* body mass index

^a^Characteristic included in Pan’s nomogram

^b^Based on the criteria of the WHO histological type (1991): *II* differentiated non-keratinizing carcinoma, *III* undifferentiated non-keratinizing carcinoma

^c^As categorized in a previous study [26]

Within a median follow-up of 60 months (range 3–108 months), 43 (8.0%), 75 (14.0%), and 74 (13.8%) patients experienced locoregional failure, distant failure, and death, respectively.

### Validation

**Fig. 1:**
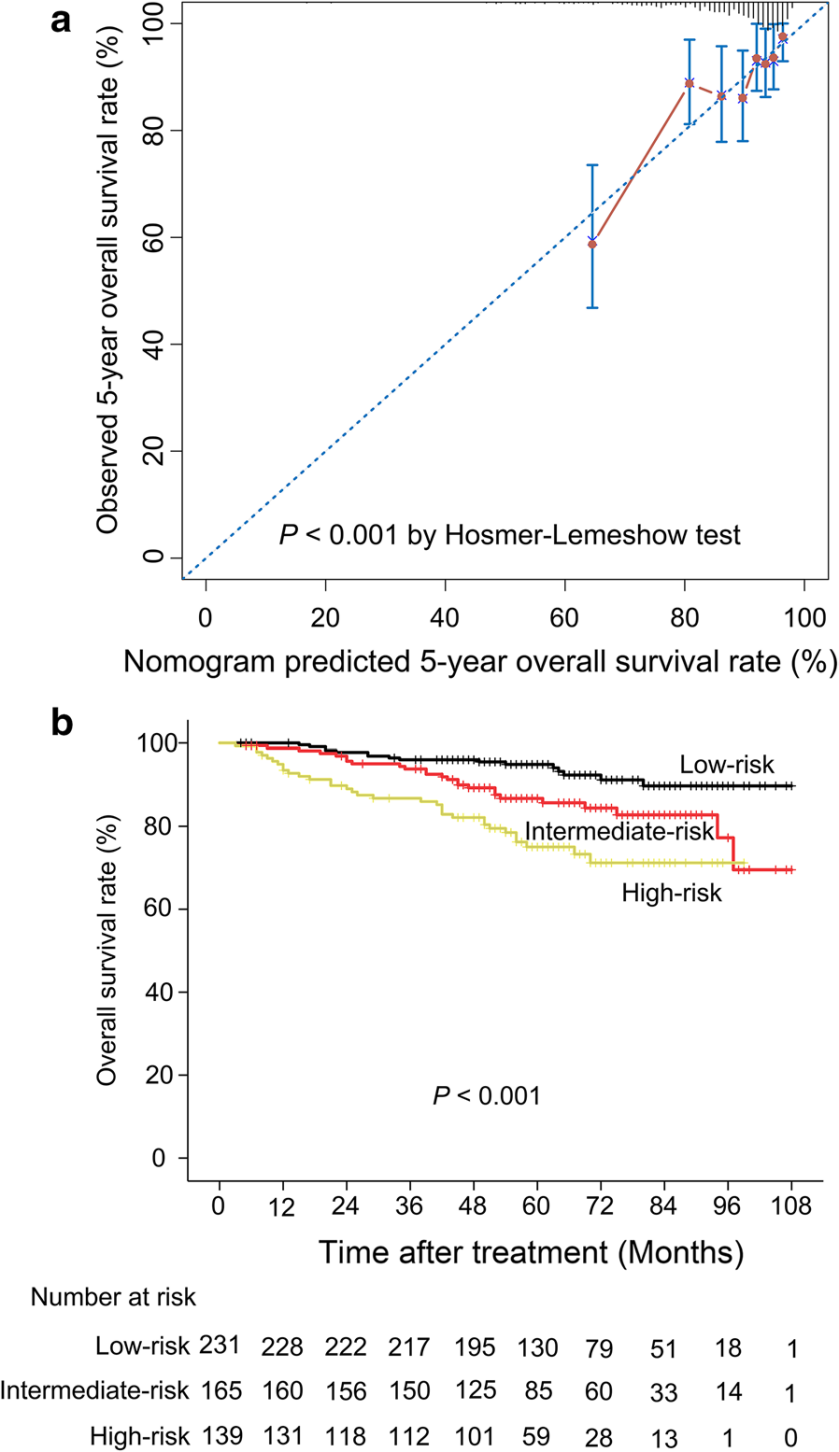
Calibration plot and survival curves for each of the investigated subgroups. **a** Calibration plot. Nomogram-predicted outcomes were stratified into three equal subgroups. For each subgroup, the average predicted probability [x-axis: nomogram-predicted 5-year overall survival (OS)] was plotted against the Kaplan–Meier calculated outcome (y-axis: observed 5-year OS). The 95% confidence intervals of the observed 5-year overall survival rate are indicated by the vertical lines. The dashed line indicates the position of an ideal nomogram. **b** Survival curve stratified by risk group

Table 2 displays the predicted and observed 5-year OS rates. Pan’s nomogram was found to underestimate the 5-year OS of the entire cohort by 8.82% (95% CI − 9.88 to − 7.77%, *P *< 0.001) in addition to the survival of each risk group. The difference between the predicted and observed 5-year OS rates were smallest among low-risk patients (− 6.88%, 95% CI − 7.22 to − 6.53%; *P *< 0.001) and largest among high-risk patients (− 13.56%, 95% CI − 15.48 to − 11.63%; *P *< 0.001). Calibration plots of the predicted *vs* observed 5-year OS rates and survival curves by stratifying risk are illustrated in Fig. 1. The Hosmer–Lemeshow test identified that the predicted and observed OS rates differed significantly from an ideal relationship between the two survival rates (*P *< 0.001).

**Table 2:** Predicted and observed 5-year overall survival rates of the different subgroups of patients

Group	No. of patients (%)	No. of deaths	5-year overall survival rate (%)			P*
			Predicted (%, SE)	Observed (%, SE)	Predicted-observed (%, 95% CI)	
Overall	535 (100.0)	74	78.46 (0.54)	87.29 (1.53)	− 8.82 (− 9.88 to − 7.77)	< 0.001
Low-risk (< 135)	231 (43.2)	16	88.04 (0.17)	94.92 (1.50)	− 6.88 (− 7.22 to − 6.53)	< 0.001
Intermediate-risk (135–160)	165 (30.8)	25	79.38 (0.24)	86.72 (2.80)	− 7.34 (− 7.81 to − 6.87)	< 0.001
High-risk (≥ 160)	139 (26.0)	33	61.46 (0.97)	75.02 (3.99)	− 13.56 (− 15.48 to − 11.63)	< 0.001

*SE* standard error, *CI* confidence interval

* One-sample *t* test

The C-index for Pan’s nomogram to predict 5-year OS was 0.710 (95% CI 0.649–0.771). When comparing the discrimination ability of Pan’s nomogram with that of other predictors, we observed that for EBV DNA (categorized), the C-index was 0.616 (95% CI 0.551–0.681), which indicated inferiority to Pan’s nomogram (*P *= 0.005). For the clinical stage determined using the 8th and 7th edition of the AJCC/UICC staging system, the C-index was 0.594 (95% CI 0.536–0.651) and 0.594 (95% CI 0.531–0.656), respectively, which was much lower as compared with that of Pan’s nomogram (both *P *< 0.001). Further, the advantage conferred by the discrimination ability achieved using Pan’s nomogram sharply decreased when compared with the combination of EBV DNA (categorized) and the clinical stage determined according to the 8th edition of the AJCC/UICC staging system (C-index 0.664, 95% CI 0.605–0.724; *P *= 0.104).

Multivariate Cox regression model using backward selection approach ultimately identified the variables, age, BMI, LDH, GTVp, and the 8th AJCC/UICC N-classification as independent prognostic factors (Table 3). Additionally, the best predictive model based on these factors achieved a marginally higher C-index (0.753, 95% CI 0.697–0.810, *P *= 0.097) when compared with that of Pan’s nomogram.

**Table 3:** Multivariate analysis of patients from the Sun Yat-sen University Cancer Center

	HR (95% CI)	*P*-value
Factors included in the best predictive model		
The 8th AJCC/UICC N-classification	1.968 (1.480–2.617)	*< 0.001*
Age (per year increase)	1.055 (1.032–1.079)	*< 0.001*
GTVp (per cc increase)	1.016 (1.009–1.023)	*< 0.001*
LDH (per IU/L increase)	1.005 (1.000–1.010)	*0.033*
BMI (per kg/m^2^ increase)	0.921 (0.854–0.995)	*0.036*
Factors absent from the best predictive model		
The 8th AJCC/UICC T-classification	1.076 (0.720–1.607)	0.721
EBV DNA (< 10^3^/10^3^–10^4^/10^4^–10^5^/10^5^–10^6^/≥ 10^6^)	1.033 (0.826–1.293)	0.774
Sex	0.759 (0.430–1.337)	0.339
Histology	0.714 (0.305–1.671)	0.437

*HR* hazard ratio, *CI* confidence interval, *AJCC* American Joint Committee on Cancer, *UICC* Union for International Cancer Control, *GTVp* gross primary tumor volume, *LDH* lactate dehydrogenase, *BMI* body mass index

## Discussion

Our findings demonstrated that Pan’s nomogram [14] underestimated the 5-year OS of patients with locoregionally advanced NPC. When the discriminatory accuracy was compared with EBV DNA, the 7th and 8th AJCC/UICC staging system, the accuracy of Pan’s nomogram was found to be superior. However, Pan’s nomogram did not demonstrate significant 5-year OS predictive ability as compared to the combination of EBV DNA together with the 8th AJCC/UICC staging system. Its discrimination performance was marginally inferior compared with that of the best predictive model, which fitted age, BMI, LDH, GTVp, and the 8th AJCC/UICC N-classification system.

The calibration ability of Pan’s nomogram derived from our database differed from the training and validation cohort of Fujian Provincial Cancer Hospital and Pamela Youde Nethersole Eastern Hospital, respectively [14]. This can be largely explained the by following. First, given that tumor stage primarily indicates tumor burden and determines treatment outcomes [28], patients with early-stage NPC usually receive only radiotherapy, whereas, for locoregionally advanced disease, concurrent chemotherapy is strongly recommended; wherein certain cases induction or adjuvant chemotherapy is also administered before or after radiotherapy. Since we included only patients with locoregionally advanced NPC, the individual treatment approaches varied by tumor stage and consequently demonstrated different treatment outcomes between the different investigated cohorts [29].

Second, the patients in our database received concurrent chemoradiotherapy alone, whereas the patients in the study by Pan et al. [14] received additional chemotherapy before or after radiotherapy. Similar to randomized controlled trials [4, 7], differences in chemotherapy approaches can also lead to differences in OS, even for tumors with similar stage. Therefore, our finding of non-accurate prediction by Pan’s nomogram was not unexpected, particularly considering the intrinsic differences in the predictions of prognosis between our independent cohort and the original training and validation cohorts [14].

In contrast, the differences among other characteristics suggest that the prediction of Pan’s nomogram was not precise enough. For example, the LDH levels of patients in our database were significantly lower compared with those of patients included in the study by Pan et al. [14] (Table 1) and the LDH level was strongly predictive of the OS of Pan’s nomogram. It is, therefore, possible that the difference in the LDH levels lowered calibration accuracy. Furthermore, a significant interaction effect was observed between the GTVp and the clinical stage according to the 8th AJCC/UICC staging system. A similar interaction effect was likely to exist when both variables were included in Pan’s nomogram during its development, for which the inferior calibration may be associated. Moreover, induction chemotherapy in clinical practice is commonly administered to patients with locoregionally advanced disease with large tumor volumes. Thus, our inclusion criteria restricting patients with locoregionally advanced disease who received concurrent chemoradiotherapy alone naturally selected patients with a relatively smaller GTVp compared with a previous report [30]. But notably, the average GTVp was not larger in our study compared with that of Pan et al. [14], which included patients with any tumor stage. So, selection bias may have exerted little effect on the underestimation of 5-year OS, because the median or average GTVp in the study by Pan et al. [14] was much larger compared with the others, in which an enlarged retropharyngeal lymph node was delineated in the GTVp [31–33].

Pan’s nomogram discriminated outcomes better compared with other single predictors such as EBV DNA and the 7th and 8th AJCC/UICC staging system. This was expected because Pan’s nomogram combined several prognostic factors with tumor stage. Unfortunately, Pan’s nomogram did not achieve significant superiority over the combination of EBV DNA and the tumor stage based on the 8th edition of the AJCC/UICC staging system. Moreover, it was marginally inferior to the model, which included independent prognostic factors such as the age, BMI, LDH, GTVp, and N-classification based on the 8th AJCC/UICC staging system.

Risk prediction programs [26, 34–37] other than Pan’s nomogram are available [14]. However, Pan’s nomogram incorporates several important and well-known clinical predictors. In particular, it is the only one developed using a cohort of patients other than those from our cancer center. However, the underestimation of OS in this external validation indicates that Pan’s nomogram cannot accurately identify authentic high-risk patients from all patients with locoregionally advanced NPC.

The limitations of this study are as follows. The lack of unified treatment approaches, chemotherapy regimens, and radiation or chemotherapy doses determined by the nature of retrospective design may, to a certain extent, bias the findings of this study. Also, due to the small sample size of patients analyzed, this could have possibly lowered the confidence of validation derived from this study. Lastly, validation by a single institution does not essentially provide a strong evidence and further large cohort, multi-institutional analysis is still required.

## Conclusions

Pan’s nomogram was observed to significantly underestimate the 5-year OS of patients with locoregionally advanced NPC. It failed to precisely identify high-risk participants for inclusion in randomized controlled trials.

## Abbreviations

AJCC: American Joint Committee on Cancer
BMI: body mass index
CI: confidence interval
C-index: concordance index
CT: computed tomography
EBV DNA: Epstein–Barr virus deoxyribonucleic acid
GTVp: gross primary tumor volume
IMRT: intensity-modulated radiotherapy
LDH: lactate dehydrogenase
MRI: magnetic resonance imaging
NPC: nasopharyngeal carcinoma
OS: overall survival
PET/CT: positron emission tomography and computed tomography
TNM: tumor, nodes and metastasis
UICC: Union for International Cancer Control
